# Aligning nutrient profiling with dietary guidelines: modifying the Nutri-Score algorithm to include whole grains

**DOI:** 10.1007/s00394-021-02718-6

**Published:** 2021-11-24

**Authors:** Katrina R. Kissock, Florent Vieux, Kevin C. Mathias, Adam Drewnowski, Chris J. Seal, Gabriel Masset, Jessica Smith, Heddie Mejborn, Nicola M. McKeown, Eleanor J. Beck

**Affiliations:** 1 School of Medicine, Faculty of Science, Medicine and Health, University of Wollongong, Wollongong, NSW 2522 Australia; 2 Illawarra Health and Medical Research Institute, Wollongong, NSW Australia; 3 MS-Nutrition, Marseille, France; 4 Skidmore College, Health and Human Physiological Sciences, Saratoga Springs, NY USA; 5 Center for Public Health Nutrition, University of Washington, Seattle, WA USA; 6 Public Health Sciences Institute, University of Newcastle, Newcastle upon Tyne, NE2 4HH UK; 7 Cereal Partners Worldwide, Prilly, Switzerland; 8 General Mills Scientific and Regulatory Affairs, Minneapolis, MN USA; 9 National Food Institute, Technical University of Denmark, Kongens Lyngby, Denmark; 10 Jean Mayer USDA Human Nutrition Research Center on Aging at Tufts University, Boston, MA USA; 11 Friedman School of Nutrition Science and Policy, Tufts University, Boston, MA USA

**Keywords:** Whole grain, Intake, Nutrient profiling, Nutri-Score, Nutrient density

## Abstract

**Purpose:**

Whole grains, generally recognised as healthy choices, are not included in most nutrient profiling systems. We tested modifications to the Nutri-Score algorithm to determine whether including whole grains would provide an improved measure of food, and overall diet quality.

**Methods:**

The whole-grain content of food, with a minimum cut-point of 25%, was added to the algorithm, following similar methods used to score other health-promoting components such as fibre. We applied and compared the original and the modified Nutri-Score to food composition and dietary intake data from Australia, France, the United Kingdom, and the United States.

**Results:**

At the food level, correlations between whole-grain content and food nutritional score were strengthened using the modified algorithm in Australian data, but less so for the other countries. Improvements were greater in grain-specific food groups. The largest shift in Nutri-Score class was from B to A (best score). At the dietary intake level, whole-diet nutritional scores for individuals were calculated and compared against population-specific diet-quality scores. With modifications, correlations with diet-quality scores were improved slightly, suggesting that the modified score better aligns with national dietary guidelines. An inverse linear relationship between whole-diet nutritional score and whole-grain intake was evident, particularly with modifications (lower whole-diet nutritional score indicative of better diet quality).

**Conclusion:**

Including a whole-grain component in the Nutri-Score algorithm is justified to align with dietary guidelines and better reflect whole grain as a contributor to improved dietary quality. Further research is required to test alternative algorithms and potentially other nutrient profiling systems.

**Supplementary Information:**

The online version contains supplementary material available at 10.1007/s00394-021-02718-6.

## Introduction

Poor quality diets are associated with a higher risk of non-communicable diseases and mortality [1]. The Global Burden of Disease study estimated that diets low in whole grains and fruit and high in sodium accounted for more than half of diet-related deaths in 2017, and that the lack of whole grains in the diet may be the primary dietary contributor to cardiovascular mortality and disability-adjusted life years (DALYs) [1]. In an effort to promote positive dietary habits, dietary guidelines across the globe provide advice on consuming more whole grains, fruit, vegetables, and legumes and limiting sodium, added sugars, and saturated fat [2]. The health benefits of whole grains may be attributed in part to cereal fibre, yet other whole-grain components, such as magnesium and polyphenols, may deliver potential health benefits [3, 4]. Dietary guidelines have encouraged the intake of whole grains, either as quantified recommendations [5, 6], or generically linked to choices within a food group [7–9]. Despite well-documented benefits and recommendations, whole-grain consumption remains low in most countries [10–15].

Nutrient profiling (NP) systems can be used to assist consumers in identifying healthier food choices. Ideally, NP systems used for front-of-pack (FOP) labelling assist consumers to make better food choices and promote adherence to dietary guidelines [16]. Most NP systems used for nutrition policy and regulation tend to focus on limiting the content of energy, saturated fat, sugar, and sodium in foods, with some systems promoting beneficial nutrients and foods such as fibre, fruit, and vegetables. However, it is clear there are gaps in the alignment of NP systems with dietary guidelines, particularly for incorporating whole grains in the diet [17]. In principle, we believe only an NP system that accounts for healthful food items including whole grain, while discouraging deleterious components will best support healthy dietary patterns.

Despite the abundance of dietary recommendations, whole grains are not routinely included in NP systems. Systems such as the Health Star Rating (Australia) and Nutri-Score (France, Belgium, Germany, Luxembourg, The Netherlands, Spain, and Switzerland) are based on the Food Standards Agency (FSA)/Ofcom model (United Kingdom (UK)). These systems are designed to encourage the intake of ‘beneficial’ components such as protein, fibre, fruit, vegetables, nuts, and legumes, while discouraging the intake of ‘detrimental’ components such as energy, saturated fat, sugar, and sodium. The whole-grain content of foods is currently not included in algorithms for these models. The Nordic Keyhole label is the only FOP NP system to include whole grain within its algorithm. Foods displaying the Keyhole label must fulfil certain conditions around the content of whole grain in addition to fat, sugar, salt, dietary fibre, fruit, and vegetables [18].

It is postulated that including whole grains in NP systems would substantially contribute to an improvement in whole-diet quality and hold relevance in encouraging and promoting greater whole-grain intake at the population level [17]. The aim of this study was to apply modifications to the Nutri-Score algorithm to include whole grain as a beneficial dietary component and to determine whether the Nutri-Score algorithm once modified would improve a theoretical measurement of food and diet quality.

## Methods

Briefly, this study involved development and preliminary analysis of modifications to the Nutri-Score algorithm to include the whole-grain content of foods as an additional beneficial component in the algorithm. We selected the Nutri-Score NP system due to current use in the European Union, with the potential to become the mandatory FOP nutrition labelling system. The original Nutri-Score algorithm and modifications were applied to food composition and dietary intake data from Australia, France, the UK, and the US as examples.

### Food composition data

Food composition data were derived from the Australian Food, Supplement and Nutrient Database (AUSNUT) 2011–2013 [19], the French CIQUAL 2013 food composition database [20], the UK National Diet and Nutrition Survey (NDNS) Nutrient Databank (years 1–6) [21], the NHANES 2015–16 Day 1 Individual Foods dataset [22], and the USDA Food Patterns Equivalents Database (FPED) 2015–2016 [23].

In the Australian context, whole-grain content of foods was obtained from the Australian whole-grain database [12, 24]. Based on AUSNUT 2011–2013, which contains 5740 food items, this database utilises a unique coding system to match foods consumed in the Australian National Nutrition and Physical Activity Survey (NNPAS) 2011–2012. For French data, the whole-grain content of foods was estimated from the Etude Individuelle Nationale des Consommations Alimentaires 2 (INCA2) recipe database [25] which provides a list and quantities of ingredients for each composite food. Each whole-grain ingredient was identified to estimate the whole-grain content of foods. For analysis of UK data, the whole-grain content of foods was obtained from the UK whole-grain database [4], based on the UK NDNS Nutrient Databank of approximately 5900 foods. For US data, whole-grain content values were obtained by converting the ounce equivalents from the FPED 2015–2016 to grams. This database converts the foods and beverages contained within the Food and Nutrient Database for Dietary Studies (FNDDS) [26] to 37 food groups and subgroups, including whole grains.

### Application of the original Nutri-Score algorithm

The Nutri-Score NP system is a five-colour FOP nutrition label derived from the UK FSA nutrient profiling system (FSA-NPS) with the nutritional score ranging from − 15 (better nutritional score) to + 40 (poorer nutritional score) [27]. For each food or beverage item, the Nutri-Score algorithm allocates up to + 10 points individually for energy (kJ), saturated fatty acids (g), total sugar (g), and sodium (mg); while allocating up to -5 points individually for protein (g) and fibre (g), and for fruit, vegetables, nuts, legumes, and walnut/rapeseed/olive oils combined (g) (FVNLO). The point-scoring systems for each individual component vary depending on classification of the food as ‘solid food’, ‘beverage’, ‘cheese’, or ‘added fat’ [28]. The overall Nutri-Score value is the sum of the scores from individual components, which is then divided into five classes of nutritional quality ranging from A (green—most healthy, lowest nutritional score) to E (red—least healthy, highest nutritional score). Although Nutri-Score is intended for individual foods, the current study calculated Nutri-Score for both individual foods (hereafter referred to as ‘food nutritional score’) and as a whole-diet nutritional score of individuals using previously validated methods [29].

The original Nutri-Score algorithm was applied to all foods and beverages within the food composition databases described above. The following food items were excluded: alcoholic beverages, beverages/powders for medical purposes, supplements, medicines, caffeine, and single vitamin/mineral/nutrient items. The Nutri-Score Frequently Asked Questions (FAQ) document was strictly followed to classify and estimate the Nutri-Score of all products [28]. Assumptions made for country-specific databases are described in Supplemental Table 1. Specifically, the Australian Health Survey–Australian Dietary Guidelines database [30], Australian nut database [31], and the AUSNUT 2011–2013 food recipe file [32] were utilised to calculate the FVNLO content of Australian foods. In the UK context, the FSA Standard Recipes Database 1992–2012 [33] was used in conjunction with the NDNS Nutrient Databank to calculate the FVNLO content of foods. For France, the INCA2 recipe database was used to estimate the amount of FVNLO in complex dishes and nutrients of foods were derived from CIQUAL 2013. For the US, the NHANES 2015–2016 Day 1 Individual Foods dataset, the corresponding 2015–2016 FNDDS ingredients dataset, and the FPED 2015–2016 were utilised to estimate FVNLO content of the foods reported consumed by individuals in the NHANES 2015–2016.

### Application of modifications to the original Nutri-Score algorithm

We considered the most recent recommendations for a whole-grain food definition from the Whole Grain Initiative (WGI) in application of the whole-grain modification to the Nutri-Score algorithm [34]. This definition, endorsed by the International Association for Cereal Science and Technology, the Healthgrain Forum, and Cereals and Grains Association, states that to be considered a whole-grain food, a food item shall contain at least 50% whole-grain ingredients based on dry weight. Furthermore, it is suggested that only foods containing a minimum of 25% whole-grain ingredients based on dry weight, may make an FOP claim on the presence of whole grain, but cannot be designated ‘whole grain’ in the product name. For inclusion of whole grain into the Nutri-Score algorithm, we applied the following steps. (1) The whole-grain percentage cut-offs outlined by the WGI were included in the beneficial points component (similar to other health-promoting nutrients and foods, e.g., fibre, fruits, and vegetables) of the Nutri-Score algorithm. Up to − 5 points were allocated using a non-linear sliding scale, to all foods containing 25–100% whole grain on a dry-weight basis (Table 1). The higher negative score allocated for ≥ 50% was used as a possible mechanism to incentivise food manufacturers to meet the ‘whole-grain food’ target. (2) Whole-grain content on a dry-weight basis was calculated for foods within each country-specific food composition database. Dry-weight calculations are published elsewhere [12]. (3) The whole-grain points’ component was only applied to food items classified as ‘solid food’. The modified Nutri-Score algorithm, like the original, was applied to the same food composition data outlined above.

**Table 1:** Summary of the whole-grain modification to Nutri-Score algorithm

Score	0	− 1	− 2	− 3	−4	− 5
Whole-grain percentage (dry weight)^a^	< 25%	≥ 25%		≥ 50%		100%

A lower nutritional score is indicative of better nutritional quality

^a^Whole-grain percentage cut-offs are derived from the Whole Grain Initiative recommendations for a whole-grain food definition

### Dietary intake data

The Australian NNPAS 2011–2012 collected dietary intake data on 12,153 participants aged 2 years and over, through two separate 24-h recalls. The first 24-h recall was conducted through face-to-face interviews with computer assistance (*n* = 12,153), while a second 24-h recall was conducted via telephone (*n* = 7735). Specific details are described elsewhere [35]. Data were analysed from the *Australian Health Survey: Nutrition and Physical Activity, 2011–2012 basic confidentialised unit record files* dataset. For the purpose of this study, only data from day 1 and adults were used (≥ 18 years, *n* = 9430 following exclusions).

For France, data on dietary intakes were obtained from the second French individual cross-sectional food consumption survey (INCA2) utilising a complex sampling design that is described elsewhere [25, 36]. The INCA2 survey aimed to estimate the amount of food and beverages consumed by individuals in a representative sample of the French population, using a 7-day open-ended food record. Only data from the adult population (18–79 years old, *n* = 2624) were used.

In the UK, the NDNS rolling programme collected data related to diet and nutrient intake in individuals aged 1.5 years and over, living in private households in the UK through a four-day food diary [37, 38]. A combined 9374 participants from years 1 to 6 (2008–2014) of the survey completed at least 3 of 4 diary days [39, 40]; only data from day 1 and those aged 18 years or over were included in this study (*n* = 4946 following exclusions).

US data were derived from the nationally representative cross-sectional NHANES 2015–2016 which collected dietary information of the non-institutionalised US population [22]. The first 24-h recall was collected in person via a validated automated multiple pass method, while a second 24-h recall was conducted via telephone. Only data recorded by adults (≥ 18 years; *n* = 5266 after exclusions) from the first 24-h recall were included in this study, consistent with a single day of data from the Australian and UK analyses.

### Estimation of whole-diet nutritional scores

The original and modified Nutri-Score algorithm was applied to national dietary intake data to determine the whole-diet nutritional score for individuals. This was calculated based on the sum of individual nutritional scores of foods by consumed energy density over total energy consumed (Calculation 1) [29]. Water and other foods/beverages with zero energy content were excluded from analyses and participant data that reported consumption of these foods/beverages only were excluded from analyses.

#### Calculation 1

$${\text{Whole-diet }}\,{\text{nutritional}}\,{\text{ score}} = \frac{{\mathop \sum \nolimits_{i = 1}^{n} {\text{FS}}_{i} E_{i} }}{{\mathop \sum \nolimits_{i = 1}^{n} E_{i} }}.$$

FS_*i*_ represents the food or beverage nutritional score, and *E*_*i*_ represents energy intake from the food or beverage. An increase in whole-diet nutritional score reflects decreasing quality from foods consumed.

### Statistical analysis

Statistical analyses were performed using Stata (StataCorp Stata Statistical Software: Release 15, 2017, College Station, TX, USA) and SAS (SAS Institute Inc: Version 9.4, Cary, NC, USA).

We compared values derived through the original and modified Nutri-Score algorithms, specifically investigating the correlation between individual components of the score (total energy, saturated fat, sugar, sodium, whole grain, fibre, protein, and FVNLO) and the final food nutritional score through Spearman’s correlations. Additionally, the changes to food nutritional score and Nutri-Score classes of all foods and those within grain-specific food groups were determined.

To identify the extent to which the original and modified Nutri-Score algorithms aligned with diet-quality scores, population-specific diet-quality scores were compared with whole-diet nutritional scores of individuals through Pearson’s correlations. These included the Healthy Eating Index for Australian Adults (HEIFA) [41], the simplified Programme National Nutrition Santé Guideline Score (PNNS-GS) 2 [42], the modified Healthy Diet Score (HDS) [43], and the US Healthy Eating Index (HEI) 2015 [44] for Australia, France, the UK, and the US, respectively. These diet-quality scores are based on dietary intake guidelines and recommendations within each country, where a higher score is reflective of better diet quality. For the modified HDS, we used Englyst fibre values based on previous dietary fibre recommendations in the UK [45, 46]. Correlations were considered significant if *P* < 0.05.

Data were converted into non-consumers and quartiles of whole-grain intake (per 10 MJ of energy intake/day) to determine the association between whole-grain intake and whole-diet nutritional score for the original and modified algorithms. Quantile analyses were performed using ANOVA with overall significance determined at *P* < 0.05. Mean diet-quality scores for whole-grain intake groups were calculated and compared against the associations between whole-grain intake and whole-diet nutritional score.

## Results

### Application of the original Nutri-Score algorithm

**Fig. 1:**
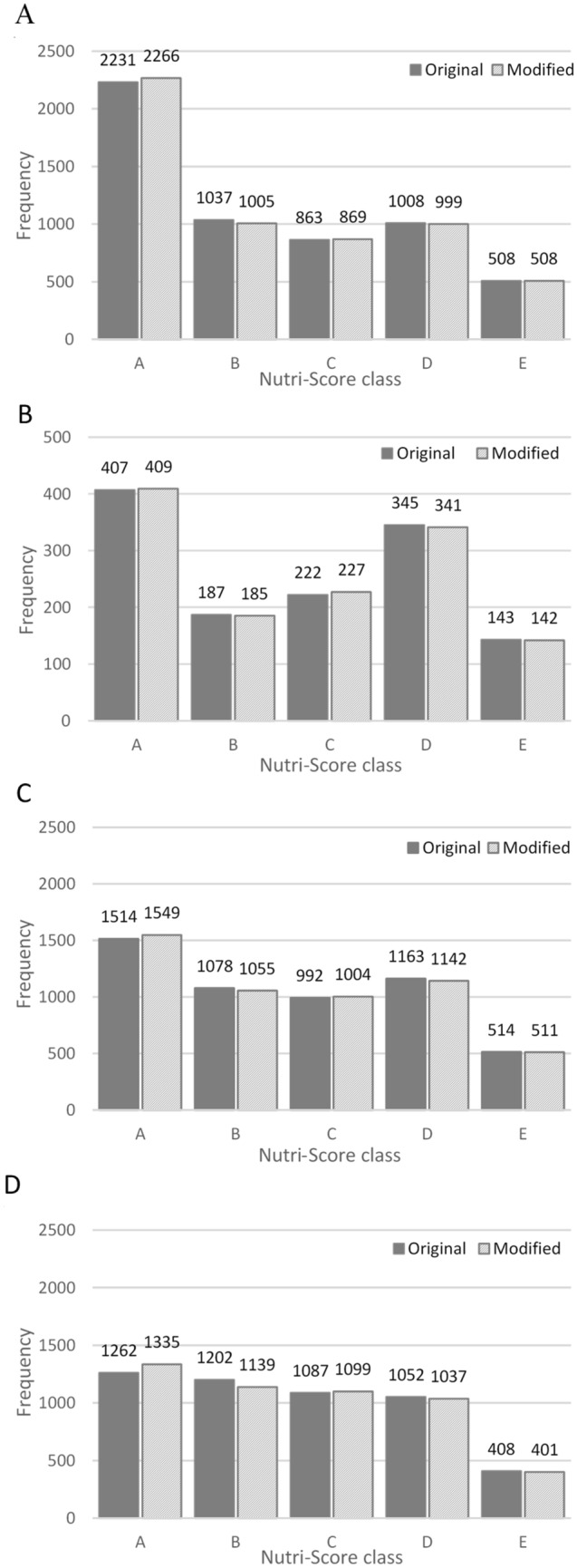
Effect of whole-grain modification to Nutri-Score class for all foods compared with the original Nutri-Score algorithm for Australia (**A**; *n* = 5647), France (**B**; *n* = 1304), the UK (**C**; *n* = 5261), and the US (**D**; *n* = 5011)^ab^. ^a^Higher Nutri-Score class denotes better nutritional quality of foods. ^b^France has a different scale for y-axis due to a minimal number of included foods

Following exclusions, 5647, 1304, 5261, and 5011 foods from Australian, French, UK and US food composition datasets were included in the country-specific analyses. Application of the original Nutri-Score algorithm exhibited similar distributions of Nutri-Score class A to E across all datasets with the highest number of foods scoring a Nutri-Score A (Fig. 1). When including all foods, correlations between content of components in the food (in g, mg, or kJ) and total nutritional score of the food indicate detrimental components, particularly energy and saturated fat, were the predominant drivers of food nutritional score (Table 2). However, there were some differences between food types (Supplemental Table 2). For example, foods in the composite ‘cereal and cereal products’ food group in Australian data, which included bread, pasta, breakfast cereal, and individual grain food products, had a correlation of 0.26 between energy content and food nutritional score, while for the ‘cereal based products and dishes’ sub-category, including biscuits, cakes, sandwiches, and other mixed dishes, the correlation was 0.87. Food groups containing both wet and dry cereal varieties, such as the ‘cereal and cereal products’ food group, tended to have a lower correlation between energy content and overall food nutritional score likely due to the large variation in energy density of foods within this food group [17]. Interestingly, positive correlations for components that would result in a better nutritional quality score, including fibre, protein, and FVNLO, were found across datasets (Table 2, Supplemental Table 2).

**Table 2:** Correlation of original and modified total food nutritional score with individual Nutri-Score component content for all foods and grain-specific food groups for each country examined

	Correlation between nutritional score and component content^abcd^												
Country	Whole grain	Fibre		Protein		FVNLO		Energy		Saturated fat		Sugar	Sodium
Australia													
All foods (*n* = 5639)^e^													
Original food nutritional score	− 0.07	− 0.19		− 0.01^ ns^		− 0.33		0.63		0.63		0.28	0.45
Modified food nutritional score	− 0.13	− 0.21		− 0.01^ ns^		− 0.32		0.60		0.63		0.27	0.45
Cereal and cereal products (*n* = 499)													
Original food nutritional score	− 0.39	− 0.30		− 0.20		0.19		0.26		0.23		0.41	0.46
Modified food nutritional score	− 0.63	− 0.37		− 0.22		0.10		0.21		0.18		0.29	0.46
Franc													
All foods (*n* = 1248)^e^													
Original food nutritional score	− 0.01^ ns^	− 0.15		0.11		− 0.51		0.62		0.60		0.29	0.14
Modified food nutritional score	− 0.05^ ns^	− 0.16		0.11		− 0.51		0.61		0.61		0.29	0.14
Breakfast cereals, bread, rusks, pasta, and rice (*n* = 52)													
Original food nutritional score	− 0.04^ ns^	0.16^ ns^		0.06^ ns^		0.21^ ns^		0.72		0.66		0.76	0.14^ ns^
Modified food nutritional score	− 0.19^ ns^	0.13^ ns^		0.05^ ns^		0.20^ ns^		0.75		0.68		0.76	0.09^ ns^
United Kingdom													
All foods (*n* = 5249)^e^													
Original food nutritional score	0.03		− 0.17		0.09		− 0.37		0.64		0.62	0.29	0.49
Modified food nutritional score	− 0.02^ ns^		− 0.18		0.08		− 0.36		0.63		0.62	0.29	0.49
Pasta, rice, bread, and breakfast cereals (*n* = 574)													
Original food nutritional score	− 0.19		− 0.24		− 0.20		0.09		0.39		0.49	0.33	0.51
Modified food nutritional score	− 0.34		− 0.26		− 0.19		0.10		0.38		0.47	0.30	0.51
United States													
All foods (*n* = 5009)^e^													
Original food nutritional score	0.05		− 0.24		0.25		− 0.53		0.66		0.61	0.25	0.46
Modified food nutritional score	− 0.02^ ns^		− 0.26		0.25		− 0.52		0.64		0.61	0.25	0.45
Cereal and cereal products (*n* = 383)													
Original food nutritional score	− 0.25		0.03^ ns^		0.02^ ns^		0.05^ ns^		0.68		0.40	0.63	0.59
Modified food nutritional score	− 0.42		0.02^ ns^		0.06^ ns^		0.05^ ns^		0.67		0.36	0.60	0.60

^a^ns; non-significant (P ≥ 0.05)

^b^Spearman’s correlation coefficients

^c^Component content units: whole grain (g dry weight); fibre (g); protein (g); FVNLO (g); energy (kJ); saturated fat (g); sugar (g); sodium (mg)

^d^Lower food nutritional scores denote better nutritional quality of foods

^e^Excludes ‘water’ items (100%, 0 kJ) (Australia *n* = 8, France *n* = 56, the UK *n* = 12, and the US *n* = 2)

Prior to inclusion of whole-grain content “points” in the modified algorithm, and for all foods, whole-grain content was inconsistently correlated with the original food nutritional score across the datasets (Table 2). Generally, for grain-specific food groups, there were more robust negative correlations; that is, higher whole-grain content was correlated with an improved food nutritional score leading to a higher, more healthful Nutri-Score class (Australia − 0.39, US − 0.25, UK − 0.19; Table 2). However, there was no correlation in the French data where a low number of whole-grain foods were included.

### Application of the modified Nutri-Score algorithm

The addition of whole grain to the Nutri-Score algorithm made little difference to the correlations between the content of individual scored components (fibre, energy, saturated fat, etc.) and food nutritional score when considering all foods (Table 2). That is, including the content of whole grain from grain-based foods is not substantial enough to change the overall nutritional score for all foods. In addition, the correlation for whole-grain content and modified Nutri-Score within all foods was weakly negative across all countries; less than − 0.10 except for Australia (− 0.19). However, as expected, the correlation for whole-grain content as a contributor to better nutritional quality of foods was strengthened in grain-specific food groups with the modified Nutri-Score algorithm (Table 2, Supplemental Table 2). This was consistent across all datasets, with improvements in correlation ranging from 61.5% to 375% improvement (difference in correlation Australia − 0.24, 61.5%; France − 0.15, 375%; UK − 0.15, 78.9%; US − 0.17, 68.0%).

**Fig. 2:**
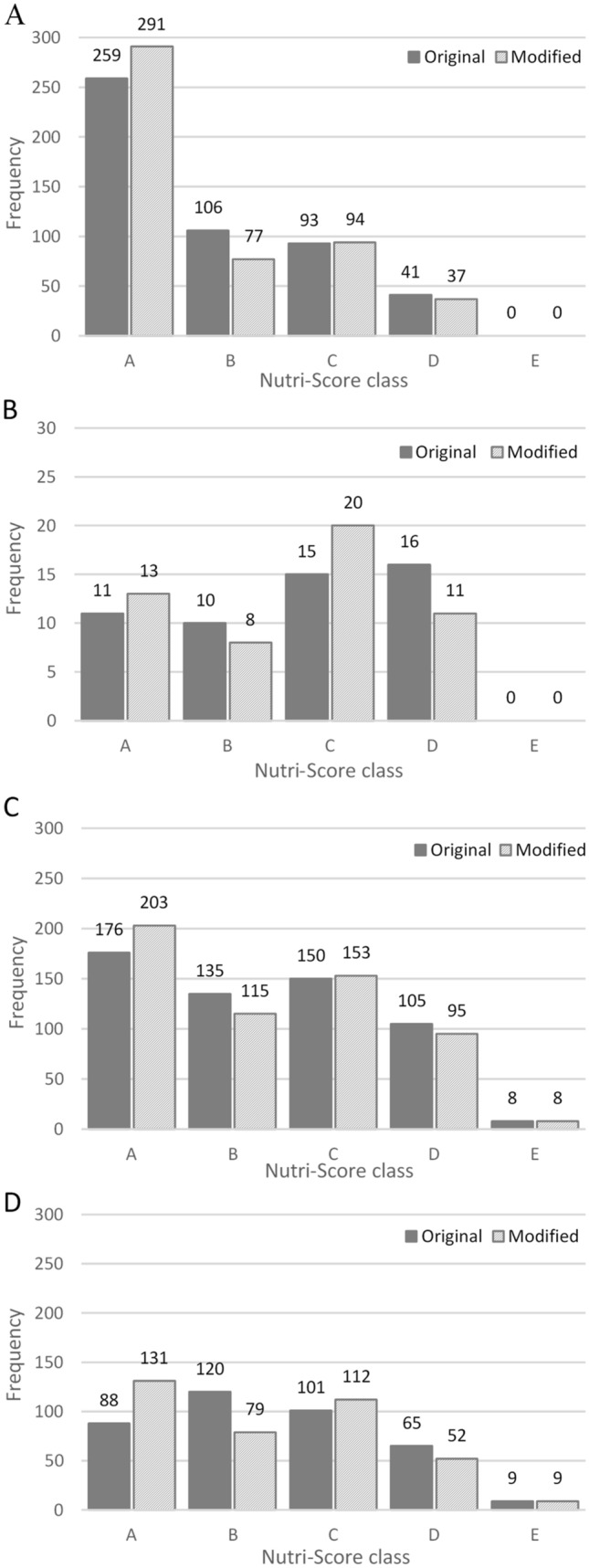
Effect of whole-grain modification to Nutri-Score class for grain-specific food groups compared with the original Nutri-Score algorithm for Australia (**A**; *n* = 499), France (**B**; *n* = 52), the UK (**C**; *n* = 574), and the US (**D**; *n* = 383)^ab^. ^a^Higher Nutri-Score class denotes better nutritional quality of foods. ^b^France has a different scale for y-axis due to a minimal number of included foods

Application of the modified Nutri-Score algorithm resulted in minimal change in Nutri-Score class across all foods (Fig. 1). However, this shift was magnified when investigating some grain-specific food groups, namely those with a higher frequency of foods containing whole grain (Fig. 2). For grain-specific food groups, the whole-grain modification predominately shifted foods from Nutri-Score class B to A, while those in Nutri-Score class C, D, and E showed limited change. For example, in Australia, 6.4% of foods in the ‘cereal and cereal products’ food group changed from Nutri-Score class B to A, while 0.8% changed from D to C and 0.6% from C to B. Foods which changed from B to A were predominately whole meal or mixed grain breads, flavoured porridge, and breakfast cereals or muesli with dried fruit. Based on the Nutri-Score individual component points (out of 10), 94% of these foods scored ≤ 5 points (≤ 27 g/100 g) for sugar with 50% scoring between 0 and 2 points (0–13.5 g/100 g); 100% scored ≤ 2 points (≤ 3 g/100 g) for saturated fat; and 88% scored ≤ 5 points (≤ 540 mg/100 g) for sodium with 66% scoring between 0–1 points (0–180 mg/100 g).

While a high percentage of grain-specific food groups containing breakfast cereals, bread, rice, and pasta had a shift in food nutritional score (for example, 36.4% in UK data to 62.5% in French data), the shift in score did not change Nutri-Score class to the same extent (the UK 7.7%; Australia 7.8%; the US 15.1%; and France 20.8%) (Table 3).

**Table 3:** Effect of whole-grain modification to food nutritional score and Nutri-Score class for all foods and grain-specific food groups compared with original Nutri-Score algorithm

	*n*^a^	Number of foods changing food nutritional score	Number of foods changing Nutri-Score class^b^
Australia			
All foods	5639	298 (5.3%)	47 (0.8%)
Cereal and cereal products	499	238 (47.7%)	39 (7.8%)
Cereal based products and dishes	914	26 (2.8%)	2 (0.2%)
Confectionary and cereal/fruit/nut/seed bars	144	14 (9.7%)	1 (0.7%)
France			
All foods	1304	26 (2.0%)	8 (0.6%)
Breakfast cereals	24	15 (62.5%)	5 (20.8%)
Bread, rusks, pasta, and rice	28	9 (32.1%)	2 (7.1%)
Sugared biscuits and cereal bars	27	1 (3.7%)	1 (3.7%)
United Kingdom			
All foods	5249	310 (5.9%)	75 (1.4%)
Pasta, rice, bread, and breakfast cereals	574	209 (36.4%)	44 (7.7%)
Buns, cakes, pastries, fruit pies, and puddings	442	22 (5.0%)	8 (1.8%)
Biscuits and snacks	186	51 (27.4%)	15 (8.1%)
United States			
All foods	5011	394 (7.9%)	112 (2.2%)
Cereal and cereal products	383	196 (51.2%)	58 (15.1%)
Cereal based products and dishes	622	79 (12.7%)	27 (4.3%)
Snack foods/cereal/fruit/bars	35	4 (11.4%)	1 (0%)

Foods containing whole grain are not solely in the grain-specific food groups listed

^a^Excludes ‘water’ items (100%, 0 kJ) (Australia *n* = 8, France *n* = 56, the UK *n* = 12, and the US *n* = 2)

^b^Food nutritional score of Nutri-Score class (foods) A: ≤ − 1, B: 0–2, C: 3–10, D: 11–18, E: ≥ 19; (beverages) A: water, B: ≤ 1, C: 2–5, D: 6–9, E: ≥ 10

### Alignment of original and modified Nutri-Score algorithms with country-specific diet-quality scores

The correlations between whole-diet nutritional score and country-specific diet-quality scores improved across all datasets with the whole-grain modification (Table 4). The change in correlation was small with only a maximum of 0.02 improvement.

**Table 4:** Correlation of whole-diet nutritional scores with country-specific diet-quality scores for original and modified Nutri-Score algorithms

	Mean whole-diet nutritional score^a^	Correlation with country-specific diet-quality score^b^
Australia (*n* = 9430)		
Original Nutri-Score algorithm	4.66	− 0.71
Modified Nutri-Score algorithm	4.38	− 0.72
France (*n* = 2624)		
Original Nutri-Score algorithm	6.62	− 0.40
Modified Nutri-Score algorithm	6.55	− 0.41
United Kingdom (*n* = 4946)^c^		
Original Nutri-Score algorithm	6.09	− 0.37
Modified Nutri-Score algorithm	5.87	− 0.38
United States (*n* = 5266)		
Original Nutri-Score algorithm	7.42	− 0.60
Modified Nutri-Score algorithm	7.28	− 0.62

^a^Lower whole-diet nutritional score indicates better nutritional quality of diet

^b^Correlation with Australia: Healthy Eating Index for Australian Adults (HEIFA); France: simplified Programme National Nutrition Santé—Guidelines Score 2 (PNNS-GS2); United Kingdom: modified Healthy Diet Score (HDS); United States: Healthy Eating Index (HEI)

^c^Modified HDS calculated using Englyst fibre values. Years 1 to 6 of UK NDNS Nutrient Databank only report Englyst fibre values. The range specified in the modified HDS account for previous dietary fibre recommendations as Englyst values

**Fig. 3:**
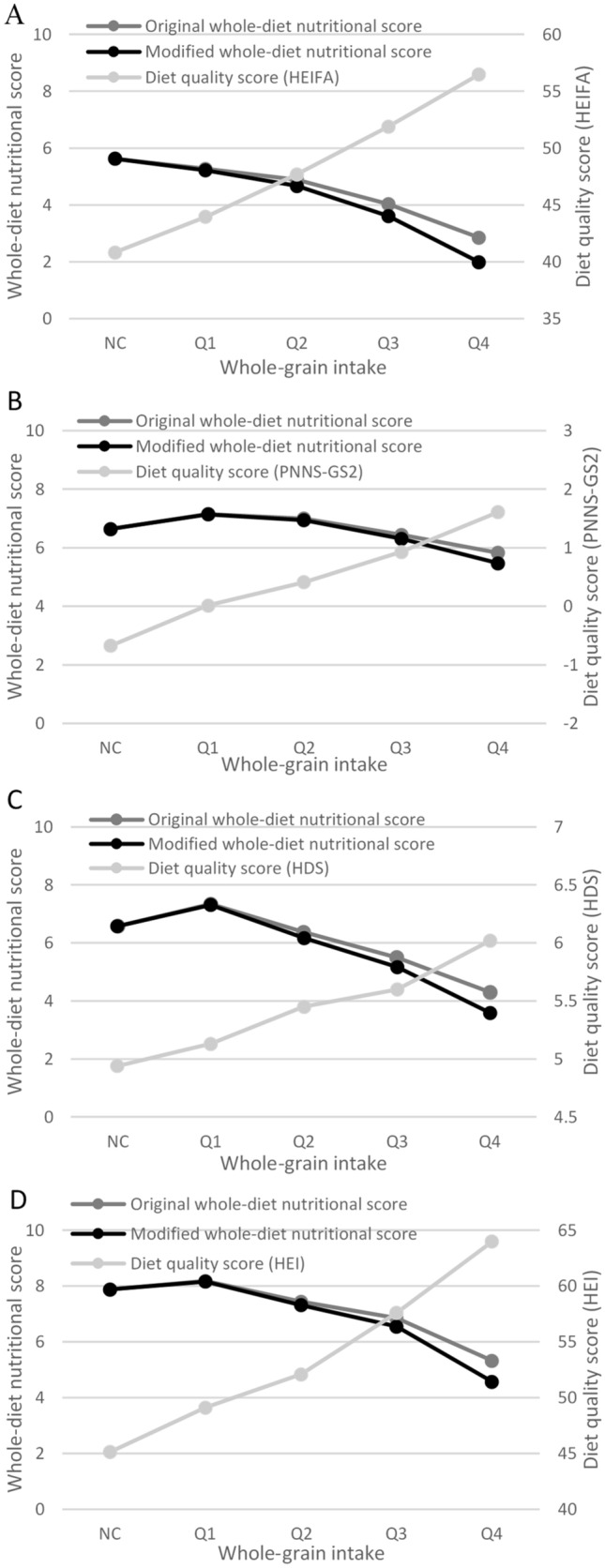
Relationship between whole-grain intake, whole-diet nutritional score for the original and modified Nutri-Score algorithm, and national diet-quality score for Australia (**A**), France (**B**), the UK (**C**) and the US (**D**)^ab^. ^a^Lower whole-diet nutritional score indicates better nutritional quality of diet. ^b^*NC* non-consumers; *HEIFA* Healthy Eating Index for Australian adults, *PNNS-GS2* Programme National Nutrition Santé—Guidelines Score 2, *HDS* Healthy Diet Score, *HEI* Healthy Eating Index

In general, there was an inverse linear trend showing better whole-diet nutritional score values with increasing daily whole-grain intake (Fig. 3). For example, in US data, non-consumers of whole grain had a mean original whole-diet nutritional score of 7.88, while the highest quartile of whole-grain intake had a mean original whole-diet nutritional score of 5.32 (lower score indicative of better diet quality) (Supplemental Table 3). With modifications to the Nutri-Score algorithm, whole-diet nutritional scores of whole-grain intake groups were incrementally improved. For example, in UK data, original compared with modified nutritional score across whole-grain consumers were Q1: 7.34 vs 7.31, Q2: 6.37 vs 6.17, Q3: 5.50 vs 5.17, Q4: 4.30 vs 3.59. In comparison, there was a positive linear trend for diet-quality scores with increasing whole-grain intake (higher diet-quality score indicative of better diet quality). Similar patterns were seen across country-specific datasets.

## Discussion

This study shows the feasibility of modifying the Nutri-Score NP algorithm with the addition of whole grain as a beneficial component, to better align with dietary guidelines recommending increased whole-grain intake. The proposed modification led to stronger correlations with national diet-quality scores and better differentiation between low and high whole-grain consumers in the four datasets analysed. These improvements, although small, are still relevant in the context of whole-grain promotion to improve intake for global health.

At the food level, the proposed modification strengthened correlations between whole-grain content of foods and more favourable Nutri-Score values. As expected, this was particularly consistent and strengthened among grain-specific food groups.

A potential argument against inclusion of whole grain in NP algorithms that do not include this food component is that cereal fibre is already captured by dietary fibre scoring in existing systems, and cereal fibre is suggested to be a key attribute to health benefits observed with higher whole-grain intakes. However, observational studies within Australia and the UK have shown associations between whole-grain intake and reductions in cardiovascular disease risk factors even after adjustment for cereal fibre [3, 4]. This suggests that in addition to fibre, other whole-grain components may contribute to beneficial health effects [47]. Here, despite what may be considered double-counting fibre with the addition of whole grain to the Nutri-Score algorithm, stronger correlations were observed for whole grain in most grain-specific food groups, compared with fibre alone. Additionally, the concept of double-counting fibre is already present in Nutri-Score and other NP systems by including foods containing fibre, such as fruits, vegetables, nuts, and legumes. Moreover, the current maximum fibre threshold in Nutri-Score is low at 4.7 g/100 g of food, and numerous whole-grain products contain higher fibre levels. For example, 31% of foods containing whole grain in US data have > 6 g/100 g of fibre. Therefore, including a whole-grain component may better emphasise the fibre content within whole-grain foods. This notion is consistent with global research showing a diet low in whole grains is attributable to higher rates of mortality and DALYs than a diet low in fibre (3 v 1 million deaths; 82 v 20 million DALYs) [1].

We chose specific cut points for whole-grain scoring to reflect recognised values determined by the WGI as relevant to promote to consumers. That is, foods with a whole-grain content of < 25% do not receive additional scoring in this algorithm. Ideally, the NP system should only promote foods that are higher in whole grain and in particular those with a very high (50–100%) whole-grain content. As such, only a minimal number of foods were affected, reflecting the relatively small proportion of high whole-grain foods in these food supplies. In addition, a large majority of foods containing whole grain were awarded Nutri-Score class A prior to modifications to the algorithm, such that improvements to Nutri-Score class could not be detected.

Limited improvements to Nutri-Score at the dietary intake level, even with modifications including whole grain, may also be due to the low level of whole-grain consumption in the four countries. For example, foods containing ≥ 25% whole grain only contribute to 3% of energy intakes in France, with 56% of individuals classified as non-consumers of these foods. However, the addition of whole grain to NP algorithms resulting in improved NP scores provides opportunities for manufacturers to promote the benefits of whole grain within products through labelling. This may increase consumer awareness and guide them towards higher and healthier whole-grain choices [48, 49]. It may also incentivise manufacturers to include more whole grain, by replacing refined with whole grain, so that more foods can be called a ‘whole-grain food’ FOP and benefit from the improved NP score.

Inclusion of whole grain in initiatives such as Nordic Keyhole label has proved beneficial. This NP scheme indicates nutritionally better food choices within food categories. Studies in Sweden and Denmark indicate that replacing non-Keyhole foods with Keyhole equivalents would increase whole-grain intake by 654% and 76%, respectively [50, 51].

At the dietary intake level, the whole-grain modification better reflected improvement of dietary quality and greater alignment with dietary guidelines. Addition of whole grain to NP systems is important to capture all factors contributing to better diet quality, allowing consistency between public health messages on individual foods (Nutri-Score and other NP) and broad messages to improve dietary quality such as dietary guidelines.

While the work here suggests that the inclusion of whole grain in NP systems may be beneficial, the implementation of such modifications may prove challenging. First, the methods used to determine whole-grain content within foods varies greatly, including classification of ‘whole grain’ as an ingredient and the lack of an analytical method to measure whole grain in foods. Some amounts of whole grain may not be included in the datasets used to calculate scores [52, 53]. Second, whole-grain labelling on food products is not consistently regulated, leading to consumer and manufacture confusion [48]. Finally, obtaining whole-grain data for third-party assessment may be difficult as the calculation of ingredient content is not often a regular practice for manufacturers. These problems are common to other components of Nutri-Score including fruit and vegetables. Prior to implementation of modifications, it is important to ensure consistency in the calculation of whole grain within foods, including whole-grain definitions, and to establish consistent regulations for whole-grain labelling that NP systems can apply.

Various limitations are present in the current study. In the French food composition database, there were far fewer foods than in other datasets, and likely greater nutritional deviation between foods, potentially leading to larger effect sizes. Similarly, a few foods containing whole grain were included, such that correlations may not be significant. Furthermore, only 1 day of dietary intake data was utilised for Australia, the UK, and the US to maximise the sample size included in analyses. This approach does not provide a reliable estimate of usual intake and as such the interpretation of the results should be based on using estimates of population intake distributions for ‘any given day’. For this particular research question, utilising a single day of dietary intake was considered sufficient as this study investigated a comparison of methods over various distributions of intakes across different populations, in which individual dietary intakes were not required to be assessed in detail.

In addition, we did not modify the Nutri-Score algorithm beyond including a whole-grain component. Before implementing the inclusion of whole grain, it would be important to perform a full review of the scoring approach, ensuring that the nutritional score received by foods is balanced within and between food categories. It may be necessary to implement a strategy to penalise foods higher in energy, sugar, sodium, and saturated fat, for example excluding whole-grain points if a specific detrimental point content threshold is met (similar to that for protein). However, analysis of Australian foods in the ‘cereal and cereal products’ food group changing from Nutri-Score class B to A identified that few foods scored well for whole grain and poorly for sugar and sodium (and none for saturated fat). As such, in any food supply, consideration of penalisation for detrimental components may only be relevant for a few foods, although such scoring is worth investigating.

Moreover, ranges of score used to identify Nutri-Score class were not altered, and foods containing substantial amounts of whole grain may not be well distinguished from those with less whole grain, particularly relevant for class A foods. Previous research found that Nutri-Score could discriminate between whole and refined foods [54], although similar algorithms on the Australian Health Star Rating showed limited potential to make this differentiation [55].

Therefore, future research should consider testing other varied modifications that achieve balance within and between food categories, such as alterations of the scoring approach for individual food groups and nutrient components of the score; changes to the thresholds that determine Nutri-Score class; or inclusion of other nutrients and food groups, including whole grain, that are grounded in dietary guidance. Further optimization of the Nutri-Score may be necessary to adequately achieve its goal of improving population-level diet quality, either through influencing consumer food choice or food product reformulations. In addition, differing country-specific diet-quality scores were included to better reflect the food supply and dietary guidelines of each country. As such, the effects of the modified Nutri-Score algorithm within each context cannot be directly compared. Some diet-quality scores (e.g., HDS) do not consider whole grain in the criteria, meaning that correlations with the modified algorithm may not be as strong.

The addition of whole grain as a beneficial component within the Nutri-Score NP algorithm improves correlations with other dietary quality markers and better differentiates individuals of low and high whole-grain consumption. While improvements to food nutritional score were minimal, improvements are still relevant to better promote whole grain given the extensive evidence on health benefits. Consistency in whole-grain ingredient and food definitions, and regulation on whole-grain labelling are all needed prior to implementation of modifications. A modification to include whole grain in NP algorithms is justified to align with dietary guidelines and better reflect whole grain as a component to measure dietary quality. This study provides a blueprint for further research to test varied modifications to Nutri-Score and other NP systems.

## Supplementary Information

Below is the link to the electronic supplementary material.

Supplementary file1 (PDF 593 KB)

## Abbreviations

*AUSNUT*: Australian Food, Supplement and Nutrient Database
*DALY*: Disability adjusted life years
*FAQ*: Frequently asked questions
*FNDDS*: Food and nutrient database for dietary studies
*FOP*: Front-of-pack
*FPED*: Food Patterns Equivalent Database
*FSA*: Food Standards Agency
*FVNLO*: Fruit, vegetables, nuts, legumes, oils
*HDS*: Healthy Diet Score
*HEI*: Healthy Eating Index
*HEIFA*: Healthy Eating Index for Australian Adults
*INCA*: Etude Individuelle Nationale des Consommations Alimentaires
*NDNS*: National Diet and Nutrition Survey
*NHANES*: National Health and Nutrition Examination Survey
*NNPAS*: National Nutrition and Physical Activity Survey
*NP*: Nutrient profiling
*PNNS-GS*: Programme National Nutrition Santé Guideline Score
*UK*: United Kingdom
*US*: United States
*WGI*: Whole Grain Initiative

## Declarations

### Availability of data and materials

Data described in the manuscript will be made available (where possible) upon request. Raw data relating to the Australian National Nutrition and Physical Activity Survey 2011–2012 and all UK data require access approval by the Australian Bureau of Statistics and UK Data Service, respectively.

### Code availability

Code used in the statistical analysis will be made available (where possible) upon request.
