# Correction to: Aligning nutrient profiling with dietary guidelines: modifying the Nutri-Score algorithm to include whole grains

**DOI:** 10.1007/s00394-021-02779-7

**Published:** 2021-12-17

**Authors:** Katrina R. Kissock, Florent Vieux, Kevin C. Mathias, Adam Drewnowski, Chris J. Seal, Gabriel Masset, Jessica Smith, Heddie Mejborn, Nicola M. McKeown, Eleanor J. Beck

**Affiliations:** 1 School of Medicine, Faculty of Science, Medicine and Health, University of Wollongong, Wollongong, NSW 2522 Australia; 2 Illawarra Health and Medical Research Institute, Wollongong, NSW Australia; 3 MS-Nutrition, Marseille, France; 4 Skidmore College, Health and Human Physiological Sciences, Saratoga Springs, NY USA; 5 Center for Public Health Nutrition, University of Washington, Seattle, WA USA; 6 Public Health Sciences Institute, University of Newcastle, Newcastle upon Tyne, NE2 4HH UK; 7 Cereal Partners Worldwide, Prilly, Switzerland; 8 General Mills Scientific and Regulatory Affairs, Minneapolis, MN USA; 9 National Food Institute, Technical University of Denmark, Kongens Lyngby, Denmark; 10 Jean Mayer USDA Human Nutrition Research Center on Aging at Tufts University, Boston, MA USA; 11 Friedman School of Nutrition Science and Policy, Tufts University, Boston, MA USA

## Correction to: European Journal of Nutrition 10.1007/s00394-021-02718-6

The article Aligning nutrient profiling with dietary guidelines: modifying the Nutri‑Score algorithm to include whole grains, written by Katrina R. Kissock, Florent Vieux, Kevin C. Mathias, Adam Drewnowski, Chris J. Seal, Gabriel Masset, Jessica Smith, Heddie Mejborn, Nicola M. McKeown and Eleanor J. Beck, was originally published electronically on the publisher’s internet portal on 24 November 2021 without open access. With the author(s)’ decision to opt for Open Choice the copyright of the article changed on 6 December 2021 to © The Author(s) 2021 and this article is licensed under a Creative Commons Attribution 4.0 International License, which permits use, sharing, adaptation, distribution and reproduction in any medium or format, as long as you give appropriate credit to the original author(s) and the source, provide a link to the Creative Commons licence, and indicate if changes were made. The images or other third party material in this article are included in the article’s Creative Commons licence, unless indicated otherwise in a credit line to the material. If material is not included in the article’s Creative Commons licence and your intended use is not permitted by statutory regulation or exceeds the permitted use, you will need to obtain permission directly from the copyright holder. To view a copy of this licence, visit http://creativecommons.org/licenses/by/4.0/.

The original article has been corrected.

