# Supplementary material for: Aligning nutrient profiling with dietary guidelines: modifying the Nutri-Score algorithm to include whole grains
Source: Eur J Nutr. 2021 Nov 24;61(1):541–53. doi: 10.1007/s00394-021-02718-6 (PMC8783881; doi:10.1007/s00394-021-02718-6)
Supplement: Supplementary file 1 — Supplementary file1 (PDF 593 KB) [file 394_2021_2718_MOESM1_ESM.pdf]

Supplementary Information – Aligning nutrient profiling with dietary guidelines: modifying the Nutri-Score algorithm to include whole grains

European Journal of Nutrition

Katrina R Kissock, Florent Vieux, Kevin C Mathias, Adam Drewnowski, Chris J Seal, Gabriel Masset, Jessica Smith, Heddie Mejborn, Nicola M. McKeown and Eleanor J Beck

Corresponding author: Eleanor Beck (eleanor_beck@uowmail.edu.au)

School of Medicine, University of Wollongong, Wollongong, NSW, 2522, Australia.

Supplemental Table 1. Assumptions made to country-specific databases with Nutri-Score application

Country	Type of assumption	Assumptions made
Australia	Classification of foods as 'solid food', 'beverage', 'added fat' or 'cheese'	• Breakfast cereal beverages, milk prepared from milk powder, infant/toddler milk/formula, meal replacement beverages, very low energy density (VLED) beverages, protein powder drinks and plant-based milk beverages (soy, rice, oat, almond milk), goat and sheep milk classified as 'solid' food due to nutritional composition and/or similarities to regular cow's milk.<br>• Cream and sour cream were classified as 'added fat'<br>• Beverages containing >80% milk (or plant-based milk) were classified as 'solid food'<br>• Where food items were not included within the AUSNUT recipe file, the ingredient list of similar products currently on the market were investigated to calculate milk and nutritional content.
	Calculation of fruit, vegetables, nuts, legumes, olive/rapeseed/walnut oil (FVNLO) content in foods	• Content of FVLO obtained from Australian Health Survey – Australian Dietary Guidelines database, while nut content was obtained from the Australian nut database.<br>• Vegetable content in tomato paste and some legumes as rehydrated values in database, therefore this value was used without additional calculations for dried products.<br>• Almond meal and tomato paste assumed to undergo minimal processing<br>• Foods described as 'deep fried' within the food name and where no recipe was available in AUSNUT recipe file, were assumed to contain no olive/rapeseed/walnut oil<br>• Where the oil ingredient listed of foods with AUSNUT recipe file is 'oil, not further defined', assumed to contain no olive/rapeseed/walnut oil<br>• Modifications applied following the Nutri-Score Frequently Asked Questions (FAQs) document<br>- Excluded nut content from pine nuts<br>- Included nut content from chestnuts<br>- Excluded vegetable content from crisps/chips<br>- Excluded starchy vegetable content (including potatoes)
France	Classification of foods as 'solid food', 'beverage', 'added fat' or 'cheese'	• Beverages containing >80% milk (or plant-based dairy alternatives) were classified as 'solid food'<br>• Coconut milk was classified as 'solid'<br>• Butter, oil, margarine, cream and other fats were classified as 'added fats'

	Calculation of fruit, vegetables, nuts, legumes, olive/rapeseed/walnut oil (FVNLO) content in foods	• Content of FVNLO was obtained from INCA2 recipe database.<br>• Following Nutri-Score FAQ scientific document, amount of dried FVNL was multiplied by 2 when dried.
United Kingdom	Calculation of nutrient composition of foods	• Calculated as average values from years 1 to 6 to account for variation between years
	Classification of foods as 'solid food', 'beverage', 'added fat' or 'cheese'	• Infant/toddler milk/formula, meal replacement beverages, plant-based milk beverages (soya, rice, oat, almond milk), goat, sheep and buffalo milk classified as 'solid' food due to nutritional composition and/or similarities to regular cow's milk.<br>• Cream and sour cream were classified as 'added fat'<br>• Beverages containing >80% milk (or plant-based milk) were classified as 'solid food'<br>• Beverage concentrates were classified as 'beverage'
	Calculation of fruit, vegetables, nuts, legumes, olive/rapeseed/walnut oil (FVNLO) content in foods	• Content of olive/rapeseed/walnut oil obtained from FSA Standard Recipe Database<br>• Modifications applied following the Nutri-Score Frequently Asked Questions (FAQs) document<br>- Calculated dried fruit and tomato puree as rehydrated value<br>- Excluded nut content from pine nuts<br>- Included nut content from chestnuts<br>- Excluded vegetable content from crisps/chips<br>- Excluded starchy vegetable content (including potatoes)
United States[ab]	Classification of foods as 'solid food', 'beverage', 'added fat' or 'cheese'	• Cottage, ricotta and cream cheeses were classified as 'cheese'<br>• Dry powder nutritional supplements were reconstituted and classified as 'solid food'<br>• Plant-based milk beverages (soy, rice, oat, almond milk), goat and sheep milk were classified as 'solid' food due to nutritional composition and/or similarities to regular cow milk.
	Calculation of whole-grain content in foods	• Whole-grain ounce equivalents provided in the 2015-2016 FPED day 1 individual food file were converted to grams using the following conversion factors: 1 ounce equivalent contained 16 grams of whole-grain flour for breads, bagels, biscuits, muffins, cakes, cookies, pancakes and waffles; 1 ounce equivalent contained 28.35 grams of grains for intact grains or grain products such as cream of wheat, barley, bulgur, millets, oats, pasta, rice, rye, quinoa, and ready-to-eat cereals

	Calculation of fruit content in foods	• Fruit cup equivalents provided in the 2015-2016 FPED day 1 individual food file were converted to grams using the following conversion factors and sequence: 1 cup equivalent contained 250 grams of fruit; all fresh fruit was given the weight (grams) of the fruit in the day 1 NHANES dietary intake file; 0 grams if fruit juice food code name did not contain "100%"; 190 grams for canned and drained; 250 grams for canned not drained; 245 grams for applesauce; 182 grams for mixed fruit salads; and 68 grams for dried fruit in products (e.g., cereal bars or RTE cereal)
	Calculation of vegetable content in foods	• Vegetable cup equivalents provided in the 2015-2016 FPED day 1 individual food file were converted to grams using the following conversion factors: 1 cup equivalent contained 118 grams for dark green vegetables. 144 grams for red or orange vegetables, 134 grams for qualifying starchy other vegetables (e.g., corn), 140 grams for other vegetables, and 175 grams for legumes (gram estimates for legumes were combined within vegetables), and 245 grams for vegetable juices.<br>• Sweet potatoes, yams, white potatoes, potato products (e.g, chips) and cassava were not classified as vegetables
	Calculation of nut content in foods	• Nut ounce equivalents provided in the 2015-2016 FPED day 1 individual food file were converted to grams using the following conversion factors: 1 ounce = ½ or (28.35/2) grams; for nut butters 1 ounce equivalent equaled 16 grams
	Calculation of olive/rapeseed/walnut oil content in foods	• Olive and canola oil content within food codes based on a recipe were calculated using the 2015-16 ingredients file.

a. Conversion factors for mixed salad fruits, dark green vegetables, red and orange vegetables, starchy other vegetables, other vegetables and legumes: Blackstone NT, Conrad Z (2020) Comparing the Recommended Eating Patterns of the EAT-Lancet Commission and Dietary Guidelines for Americans: Implications for Sustainable Nutrition. Curr Dev Nutr 4(3):nzaa015. https://doi.org/10.1093/cdn/nzaa015

b. All other conversion factors: Food Patterns Equivalents Database 2015-2016: Methodology and User Guide. https://www.ars.usda.gov/ARSUserFiles/80400530/pdf/fped/FPED_1516.pdf

Supplemental Table 2. Correlation of original and modified total food nutritional score with individual Nutri-Score component content for all foods and grain-specific food groups for each country examined

	Correlation between nutritional score and component content[abcd]							
	Whole grain	Fibre	Protein	FVNLO	Energy	Saturated fat	Sugar	Sodium
**Australia**								
**All foods (n=5639)[e]**								
Original food nutritional score	-0.07	-0.19	-0.01^{ns}	-0.33	0.63	0.63	0.28	0.45
Modified food nutritional score	-0.13	-0.21	-0.01^{ns}	-0.32	0.60	0.63	0.27	0.45
**Cereal and cereal products (n=499)**								
Original food nutritional score	-0.39	-0.30	-0.20	0.19	0.26	0.23	0.41	0.46
Modified food nutritional score	-0.63	-0.37	-0.22	0.10	0.21	0.18	0.29	0.46
**Cereal based products and dishes (n=914)**								
Original food nutritional score	-0.31	-0.07	-0.24	-0.41	0.87	0.84	0.71	0.18
Modified food nutritional score	-0.33	-0.07	-0.25	-0.41	0.87	0.84	0.71	0.18
**Confectionary and cereal/fruit/nut/seed bars (n=144)**								
Original food nutritional score	-0.51	-0.50	-0.14^{ns}	-0.37	0.53	0.73	0.55	0.10^{ns}
Modified food nutritional score	-0.53	-0.50	-0.14^{ns}	-0.37	0.53	0.73	0.55	0.09^{ns}
**France**								
**All foods (n=1248)[e]**								
Original food nutritional score	-0.01^{ns}	-0.15	0.11	-0.51	0.62	0.60	0.29	0.14
Modified food nutritional score	-0.05^{ns}	-0.16	0.11	-0.51	0.61	0.61	0.29	0.14
**Breakfast cereals (n=24)**								
Original food nutritional score	-0.06^{ns}	0.05^{ns}	-0.14^{ns}	0.07^{ns}	0.62	0.68	0.45	0.05^{ns}
Modified food nutritional score	-0.20^{ns}	-0.06^{ns}	-0.18^{ns}	-0.06^{ns}	0.63	0.62	0.53	-0.02^{ns}
**Bread, rusks, pasta and rice (n=28)**								
Original food nutritional score	-0.35^{ns}	0.30^{ns}	0.57	0.37^{ns}	0.70	0.53	0.65	0.52
Modified food nutritional score	-0.55	0.25^{ns}	0.55	0.40	0.70	0.58	0.66	0.42
**Sugared biscuits and cereal bars (n=27)**								
Original food nutritional score	-0.15^{ns}	-0.33^{ns}	0.14^{ns}	-0.44	0.70	0.82	-0.25^{ns}	0.10^{ns}
Modified food nutritional score	-0.15^{ns}	-0.33^{ns}	0.14^{ns}	-0.44	0.70	0.82	-0.25^{ns}	0.11^{ns}
**United Kingdom**								
**All foods (n=5249)[e]**								
Original food nutritional score	0.03	-0.17	0.09	-0.37	0.64	0.62	0.29	0.49
Modified food nutritional score	-0.02^{ns}	-0.18	0.08	-0.36	0.63	0.62	0.29	0.49

**Pasta, rice, bread and breakfast cereals (n=574)**								
Original food nutritional score	-0.19	-0.24	-0.20	0.09	0.39	0.49	0.33	0.51
Modified food nutritional score	-0.34	-0.26	-0.19	0.10	0.38	0.47	0.30	0.51
**Buns, cakes, pastries, fruit pies and puddings (n=442)**								
Original food nutritional score	-0.05[ns]	0.18	0.24	-0.09[ns]	0.85	0.81	0.58	0.57
Modified food nutritional score	-0.10	0.16	0.23	-0.09[ns]	0.84	0.80	0.58	0.56
**Biscuits and snacks (n=186)**								
Original food nutritional score	-0.37	-0.66	-0.38	-0.10[ns]	0.56	0.82	0.44	0.17
Modified food nutritional score	-0.44	-0.69	-0.40	-0.09[ns]	0.56	0.81	0.44	0.15
**United States**								
**All foods (n=5009)**[e]								
Original food nutritional score	0.05	-0.24	0.25	-0.53	0.66	0.61	0.25	0.46
Modified food nutritional score	-0.02[ns]	-0.26	0.25	-0.52	0.64	0.61	0.25	0.45
**Cereal and cereal products (n=383)**								
Original food nutritional score	-0.25	0.03[ns]	0.02[ns]	0.05[ns]	0.68	0.40	0.63	0.59
Modified food nutritional score	-0.42	0.02[ns]	0.06[ns]	0.05[ns]	0.67	0.36	0.60	0.60
**Cereal based products and dishes (n=622)**								
Original food nutritional score	-0.19	0.07[ns]	0.07[ns]	-0.47	0.83	0.80	0.72	0.40
Modified food nutritional score	-0.25	0.05[ns]	0.08	-0.45	0.82	0.80	0.73	0.39
**Snack foods/cereal/fruit/bars (n=35)**								
Original food nutritional score	-0.43	-0.63	0.03[ns]	-0.01[ns]	0.66	0.60	0.19[ns]	-0.002[ns]
Modified food nutritional score	-0.48	-0.62	0.07[ns]	0.02[ns]	0.67	0.57	0.21[ns]	-0.006[ns]

a. ns; non-significant (P≥0.05)
b. Spearman's correlation coefficients
c. Component content units: whole grain (g dry weight); fibre (g); protein (g); FVNLO (g); energy (kJ); saturated fat (g); sugar (g); sodium (mg)
d. Lower food nutritional scores denote better nutritional quality of foods
e. Excludes 'water' items (100%, 0kJ) (Australia n=8, France n=56, the UK n=12, the US n=2)

Supplemental Table 3. Original and modified whole-diet nutritional score and diet-quality score across non-consumers and quartile categories of whole-grain intake[a]

	Whole-grain intake					ANOVA p-value	P-value linear trend
	Non-consumers	Q1	Q2	Q3	Q4		
**Australia**							
n	2729	1676	1675	1675	1675		
Whole-grain intake: median g/10MJ/day (range)	0	10.9 (0.0-23.6)	35.6 (23.6-47.8)	64.1 (47.9-83.7)	113.3 (83.8-408.6)		
Diet-quality score: mean (SEM)[b]	40.82 (0.28)	43.96 (0.35)	47.70 (0.36)	51.88 (0.36)	56.48 (0.34)		
Original whole-diet nutritional score: mean (SEM)[c]	5.63 (0.07)	5.27 (0.09)	4.89 (0.09)	4.03 (0.09)	2.85 (0.09)	<0.001	<0.001
Modified whole-diet nutritional score: mean (SEM)[c]	5.63 (0.07)	5.22 (0.09)	4.67 (0.09)	3.61 (0.09)	1.99 (0.09)	<0.001	<0.001
**France**							
n	1332	324	322	323	323		
Whole-grain intake median g/10MJ/day (range)	0	2.9 (0.0-6.5)	10.8 (6.5-15.8)	23.1 (15.9-35.4)	53.5 (35.4-329.9)		
Diet-quality score: mean (SEM)[b]	-0.67 (0.10)	0.01 (0.19)	0.41 (0.19)	0.93 (0.18)	1.61 (0.19)		
Original whole-diet nutritional score: mean (SEM)[d]	6.64 (0.06)[A]	7.15 (0.11)[B]	7.00 (0.11)[AB]	6.44(0.11)[A]	5.83 (0.12)	<0.001	<0.001
Modified whole-diet nutritional score: mean (SEM)[d]	6.64 (0.06)[A]	7.14 (0.11)[B]	6.94 (0.11)[AB]	6.31 (0.11)[A]	5.47 (0.12)	<0.001	<0.001
**United Kingdom**							
n	1505	861	860	860	860		
Whole-grain intake median g/10MJ/day (range)	0	9.6 (0.1-22.5)	33.1 (22.6-43.9)	56.1 (44.0-73.6)	100.8 (73.7-413.3)		
Diet-quality score: mean (SEM)[b]	4.94 (0.04)	5.13 (0.06)	5.45 (0.06)	5.60 (0.06)	6.02 (0.06)		
Original whole-diet nutritional score: mean (SEM)[d]	6.58 (0.08)[A]	7.34 (0.11)	6.37 (0.11)[A]	5.50 (0.11)	4.30 (0.11)	<0.001	<0.001
Modified whole-diet nutritional score: mean (SEM)[c]	6.58 (0.08)	7.31 (0.11)	6.17 (0.11)	5.17 (0.11)	3.59 (0.11)	<0.001	<0.001
**United States**							
n	2666	650	650	650	650		
Whole-grain intake median g/10MJ/day (range)	0	8.3 (0.2-14.5)	23.2 (14.5-32.9)	45.4 (33.0-64.5)	95.4 (64.5-372.1)		
Diet-quality score: mean (SEM)[b]	45.13 (0.23)	49.11 (0.48)	52.09 (0.48)	57.60 (0.50)	63.99 (0.52)		
Original whole-diet nutritional score: mean (SEM)[d]	7.88(0.08)[A]	8.18(0.13)[A]	7.44(0.14)[AB]	6.85(0.14)[B]	5.32(0.14)	<0.001	<0.001
Modified whole-diet nutritional score: mean (SEM)[d]	7.88(0.08)[A]	8.16(0.13)[A]	7.32(0.14)	6.55(0.14)	4.57(0.15)	<0.001	<0.001

a. Lower whole-diet nutritional score indicates better nutritional quality of diet
b. Diet-quality score possible range: 0 to 100 (Australia), -17 to 13.5 (France), 0 to 14 (UK) and 0 to 100 (US)
c. All means across row are significantly different ($p<0.005$; Bonferroni correction for multiple comparisons)
d. Means sharing the same letter across rows are not significantly different ($P \geq 0.005$; Bonferroni correction for multiple comparisons)
